# Tunable properties of (Ho_x_Y_1-x_)_2_SiO_5_ as damage self-monitoring environmental/thermal barrier coating candidates

**DOI:** 10.1038/s41598-018-36883-2

**Published:** 2019-01-23

**Authors:** Zhilin Tian, Liya Zheng, Wanpeng Hu, Luchao Sun, Jie Zhang, Jingyang Wang

**Affiliations:** 1 High-performance Ceramics Division, Shenyang National Laboratory for Materials Science, Institute of Metal Research, Chinese Academy of Sciences, 110016 Shenyang, China; 2 University of Chinese Academy of Sciences, Beijing, 100049 China; 3 School of Materials Science and Engineering, University of Science and Technology of China, Hefei, 230026 China

## Abstract

RE_2_SiO_5_ with low thermal conductivity, compatible thermal expansion coefficients and excellent high-temperature reliability in harsh environments are excellent candidates as advanced environmental/thermal barrier coating materials for high-efficiency gas turbine engines. A series of rare earth silicates (Ho_x_Y_1-x_)_2_SiO_5_ are designed and their properties are comprehensively investigated in this paper. Through doping Ho into Y_2_SiO_5_, the thermal conductivity of Y_2_SiO_5_ is significantly decreased and the thermal expansion coefficient is also optimized closer to Si-based ceramics. High-temperature elastic stiffness and bending strength are increased with the enhancing of Ho content. Most important, doping Ho element provides (Ho_x_Y_1-x_)_2_SiO_5_ with tunable luminescence characteristic. (Ho_x_Y_1-x_)_2_SiO_5_ exhibit green, to yellow-green, then to orange-red luminescence color with increased Ho concentration. The results show that they can be used as damage self-monitoring environmental/thermal barrier coating materials for Si-based ceramics.

## Introduction

Gas turbine engines have benefited from decades of development of nickel-based superalloys, however, operating temperatures are now reaching limits posed by the melting temperatures of these materials^1^. Nowadays, higher operating temperatures are pursuing to achieve better efficiency and a reduction in environmentally harmful byproducts. Si-based ceramics such as SiC and SiC_f_/SiC ceramic matrix composites are increasingly used as the hot-section components in gas turbine engines due to their excellent high-temperature properties. When exposed to high-speed water vapor they are subjected to severe recession and environmental barrier coating (EBC) materials are critically needed to protect them^2^. Further increase in operating temperature, thermal barrier coating (TBC) is essential to allow a steep temperature gradient across it to lower the temperature of the matrix ceramics that for increasing the lifetime and efficiency of gas turbine engines^2^. Therefore, an integrated environmental/thermal barrier coating (ETBC) material with the functions of corrosion resistance and thermal insulation is highly desirable. Rare earth (RE) silicates have been demonstrated to be the third generation of EBC for Si-based ceramics due to their excellent corrosion resistance to water vapor and molten silicates^3,4^. Among them, Y_2_SiO_5_ is a promising EBC candidate with its merits of small density, low cost, abundant in the natural source, and relatively good thermal and mechanical properties^3,5^. Previous work showed that thermal conductivity of Y_2_SiO_5_ is relatively higher than its RE_2_SiO_5_ counterparts and it needs modification on its performances for thermal insulation applications^5^. In addition, thermal expansion coefficients of Y_2_SiO_5_ is relatively larger than those of Si-based ceramics that may cause the exfoliation of coating material under longtime heating and cooling cycles. Consequently, it is urgent to lower the thermal conductivity and thermal expansion coefficients and improve the high-temperature mechanical properties of Y_2_SiO_5_ to realize its application as ETBC for Si-based ceramics.

In this work, the doping method was applied to improve the thermal and mechanical properties of Y_2_SiO_5_. Our prior work revealed that Ho can endow RE_2_SiO_5_ with excellent thermal and mechanical properties^5^. Therefore, Ho doping was adopted to modify the properties of Y_2_SiO_5_. (Ho_x_Y_1-x_)_2_SiO_5_ solid solutions were prepared using the hot pressing method. Thermal conductivities, thermal expansion coefficients, bending strengths, elastic moduli and internal friction of (Ho_x_Y_1-x_)_2_SiO_5_ were measured from room to high temperatures. The thermal conductivity of Y_2_SiO_5_ can be significantly reduced by introducing Ho into the crystal lattice and its high-temperature stiffness and bending strength can also be improved. In addition, (Ho_x_Y_1-x_)_2_SiO_5_ were found to exhibit green, yellow-green and orange-red colors with various Ho doping concentration. This may contribute to damage self-detecting when the solid solution is adopted in the multi-layered or gradient ETBC coating. The present results show that (Ho_x_Y_1-x_)_2_SiO_5_ solid solutions are promising damage self-monitoring ETBC candidates.

## Experiment

### Sample preparation

Bulk (Ho_x_Y_1-x_)_2_SiO_5_ (x = 0, 1/3, 2/3, and 1) samples were prepared by hot pressing method. At first, Y_2_O_3_, Ho_2_O_3,_ and SiO_2_ powders were mixed according to stoichiometric ratios by ball milling for 24 h. The obtained slurry was dried at 60 °C for 24 h. The mixture was annealed at 1550 °C for 1 h to synthesize (Ho_x_Y_1-x_)_2_SiO_5_ powders. Dense (Ho_x_Y_1-x_)_2_SiO_5_ ceramics were hot pressed at 1600 °C. The densities of the as-synthesized samples were determined by Archimedes method and the densities of as-synthesized (Ho_x_Y_1-x_)_2_SiO_5_ samples were more than 94% of the theoretical values. The phase compositions of samples were identified using X-ray diffractometer with Cu*Kα* radiation (D/max-2400, Rigaku, Tokyo, Japan). Microstructures were observed with a SUPRA 55 scanning electron microscope (LEO, Oberkochen, Germany).

### Mechanical properties measurements

The dynamic Young’s modulus and internal friction of the samples were evaluated through an impulse excitation technique using samples with dimensions of 3 mm × 15 mm × 40 mm. The samples were measured in a graphite furnace RFDA-HTVP 1750C (IMCE, Diepenbeek, Belgium) at a heating rate of 4 °C/min in an Ar atmosphere. Vibration signals captured by a laser vibrometer were analyzed by a resonance frequency and damping analyzer, and Young’s modulus was calculated from the flexural resonant frequency, according to ASTME 1876–97.

Bending strength was measured in a universal testing machine (CMT4204, SANS, Shenzhen, China) using samples with dimensions of 3 mm × 4 mm × 36 mm. The three-point bending method with a crosshead speed of 0.5 mm/min was applied, and three samples for each (Ho_x_Y_1-x_)_2_SiO_5_ specimen were measured.

### Thermal properties measurements

Experimental thermal conductivity was determined from the measurements of thermal diffusivity *a*, heat capacity *C*_*p*_, and density *ρ*:

1 $$\kappa =a{C}_{p}\rho $$

Thermal diffusivities were measured using a laser flash analyzer (Netzsch LFA 457, Bavaria, Germany) in an argon atmosphere from room temperature to 1273 K. Both sides were sprayed with a thin layer of colloidal graphite to ensure complete and uniform absorption of the laser pulse. Isobaric heat capacity *C*_*p*_ of (Ho_x_Y_1-x_)_2_SiO_5_ were obtained from literature data in terms of their constituent binary oxides (Y_2_O_3_, Ho_2_O_3,_ and SiO_2_) by Neumann-Kopp rule^6^.

Thermal expansion coefficients were determined by temperature-dependent changes in the length of specimens from room temperature to 1473 K by using a vertical high-temperature optical dilatometer (ODHT, Modena, Italy). The dimensions of the samples are 3 × 4 × 14 mm^3^.

### Luminescence properties measurements

UV-Vis absorption spectral studies were carried out by UV-VIS-NIR spectrophotometer (SolidSpec-3700DUV, Shimazu, Japan). Photoluminescence (PL) spectra were measured on a steady-state fluorescence spectrophotometer (Fluorolog-3-TAU, Horiba Jobin Yvon, France).

## Results and Discussion

**Figure 1:**
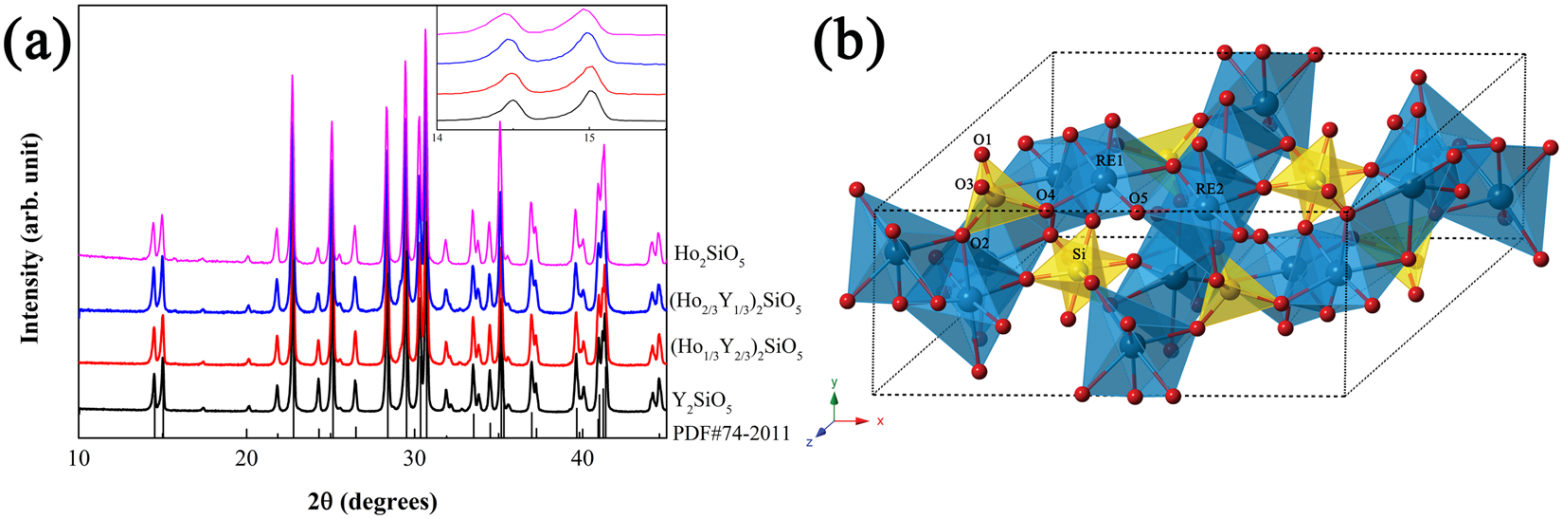
(**a**) XRD patterns of as-sintered (Ho_x_Y_1-x_)_2_SiO_5_ samples and (**b**) crystal structure of X2-RE_2_SiO_5_.

Figure 1a shows the X-ray diffraction (XRD) patterns of as-sintered (Ho_x_Y_1-x_)_2_SiO_5_ samples. They are consistent with the standard XRD spectrum of X2-Y_2_SiO_5_ (ICCD PDF No. 74-2011). All the samples are pure enough without detectable impurities. With the increase of Ho concentration, XRD patterns show a blue shift (inset in Fig. 1a) and it proves that a number of Y atoms were substituted by Ho atoms in the lattice of X2-Y_2_SiO_5_. RE_2_SiO_5_ has two polymorphs, both of which are monoclinic with space groups *P*2_1_/*c* for larger RE elements (X1 phase) and *C*2/*c* for smaller RE elements (X2 phase). The coordination of RE for the X1 and X2 phase are 9, 7 and 7, 6 respectively^7^. Y_2_SiO_5_ possesses both X1 and X2 phases depending on the fabrication temperature. In this work, the as-prepared samples belong to X2-RE_2_SiO_5_. Figure 1b is the crystal structure of X2-RE_2_SiO_5_ and it contains two RE atomic sites, one Si atomic sites, and five oxygen atomic sites. The ionic radius of Ho^3+^ and Y^3+^ are 0.894 and 0.88 Å (six coordination)^8^, respectively and their difference is within 15% which allowed the materials being substitutional solid solutions^9^. The doped Ho occupies the two RE atomic sites and the mismatch of ionic radii of Ho^3+^ and Y^3+^ results in enhanced lattice parameters causing the blue shift of XRD patterns.

**Figure 2:**
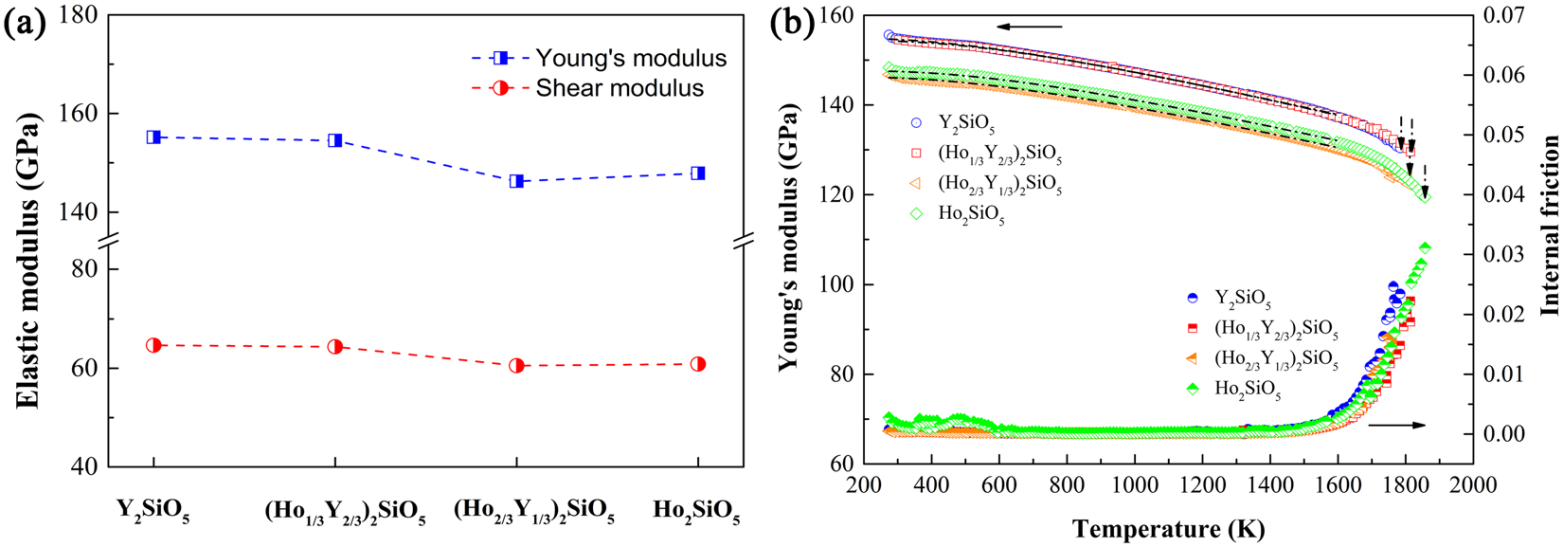
(**a**) Young’s moduli and shear moduli; (**b**) temperature dependent Young’s moduli and internal friction of (Ho_x_Y_1-x_)_2_SiO_5_.

Figure 2a presents Young’s moduli and shear moduli of (Ho_x_Y_1-x_)_2_SiO_5_. Both of them decrease with the increase of Ho concentration. High-temperature Young’s moduli and internal friction were measured and shown in Fig. 2b. The Young’s moduli decrease linearly from room temperature to 1600 K. Then, they drop relatively faster accompanied by an exponential increase of internal friction. The quick decrease of Young’s moduli and increase of internal friction suggest the stiffness softening of the samples. The Young’s moduli of Y_2_SiO_5_ and (Ho_1/3_Y_2/3_)_2_SiO_5_ are close in magnitudes and those of (Ho_2/3_Y_1/3_)_2_SiO_5_ and Ho_2_SiO_5_ are nearly the same. Ho_2_SiO_5_ has excellent high-temperature stiffness retention compared with that of Y_2_SiO_5_ because its Young’s modulus can be measured up to 1857 K. For Y_2_SiO_5_, Young’s modulus can only be measured up to 1784 K and no signal can be detected at higher temperature due to weak response and strong noise. When doping with Ho, the high-temperature stiffness can be obviously improved. The Young’s moduli of doped samples can be retained at a higher temperature than that of Y_2_SiO_5_. Temperature dependence of Young’s modulus for crystalline materials can be attributed to the anharmonic effects of lattice vibrations and decreases in bond strengths at elevated temperature.

**Figure 3:**
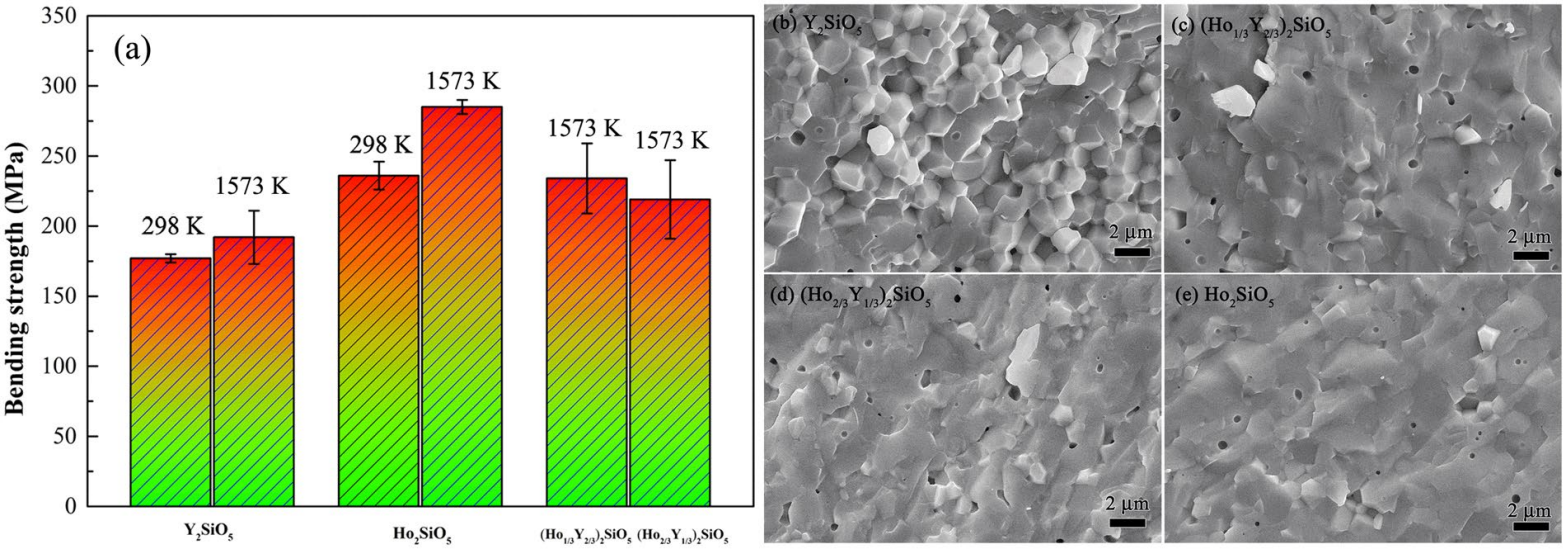
(**a**) Bending strengths of (Ho_x_Y_1-x_)_2_SiO_5_ at room and high temperatures; (**b**–**e**) morphologies of the fracture surface of (Ho_x_Y_1-x_)_2_SiO_5_ after bending strength at 1573 K.

As the Ho doping effectively improves the high-temperature stiffness, the high temperature bending strength of (Ho_x_Y_1-x_)_2_SiO_5_ were measured and exhibited in Fig. 3a. Both room temperature and high temperature bending strengths of Ho_2_SiO_5_ are higher than that of Y_2_SiO_5_. When doping with Ho, bending strengths of (Ho_1/3_Y_2/3_)_2_SiO_5_ and (Ho_2/3_Y_1/3_)_2_SiO_5_ at 1573 K are 234 ± 25 and 219 ± 28 MPa, respectively, they are higher than that of Y_2_SiO_5_ and lower than that of Ho_2_SiO_5_. The force constant is proportional to the strength of a chemical bond. Luo *et al*. and Li *et al*.^10,11^ calculated the average interatomic force constants in RE_2_SiO_5_. Their theoretical results showed that average force constants of Y-O and Ho-O are 2.6 and 6.5 eV/Å^2^ in Y_2_SiO_5_ and Ho_2_SiO_5_, respectively. Therefore, incorporating Y-O bond with smaller force constant corresponds to the relatively lower strengths of (Ho_1/3_Y_2/3_)_2_SiO_5_ and (Ho_2/3_Y_1/3_)_2_SiO_5_ than that of Ho_2_SiO_5_.

Therefore, high-temperature mechanical properties can be improved through doping with Ho. To investigate the mechanism of high-temperature enhancement, the morphologies of the fracture surface after bending strength test at 1573 K are illustrated in Fig. 3b–e. Grains can be obviously found in the fracture surface of Y_2_SiO_5_ indicating an intergranular fracture. On the contrary, (Ho_1/3_Y_2/3_)_2_SiO_5_, (Ho_2/3_Y_1/3_)_2_SiO_5_ and Ho_2_SiO_5_ present mostly transgranular fracture. As we know, grain boundaries are weakly bounded interfaces where fractures usually generate. In transgranular fracture, cracks go through the crystals instead of only along grain boundaries. Therefore, Ho doping can modify the high-temperature mechanical properties through the change of fracture modes at elevated temperature.

**Figure 4:**
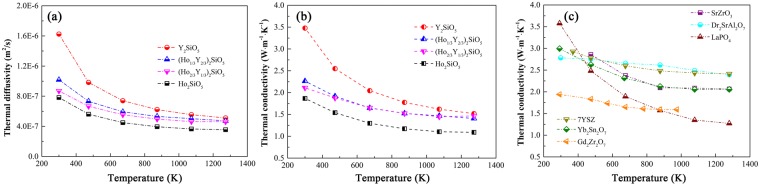
(**a**) Thermal diffusivities of (Ho_x_Y_1-x_)_2_SiO_5_ and thermal conductivities of (**b**) (Ho_x_Y_1-x_)_2_SiO_5_ and (**c**) several TBC candidates.

Thermal conductivity is one of the key parameters for ETBC candidates. Figure 4a is the temperature dependent thermal diffusivities of (Ho_x_Y_1-x_)_2_SiO_5_. They decrease with the increase of temperature and then gradually approach steady at high temperature. Thermal diffusivity of Y_2_SiO_5_ is much higher than that of Ho_2_SiO_5_ and the values could be decreased with the rise of Ho content. With the increase of temperature, the gap of thermal diffusivity between Y_2_SiO_5_ and Ho_2_SiO_5_ is reduced. Thermal conductivity can be obtained based on Eq. 1 and the results are shown in Fig. 4b. Thermal conductivities of (Ho_1/3_Y_2/3_)_2_SiO_5_ and (Ho_2/3_Y_1/3_)_2_SiO_5_ are lower than that of Y_2_SiO_5_. The room temperature thermal conductivity of (Ho_1/3_Y_2/3_)_2_SiO_5_ is slightly higher than that of (Ho_2/3_Y_1/3_)_2_SiO_5_. They tend to be close as temperature increases. Callaway suggested that the thermal conductivities of a solid containing defects can be calculated by^12^:

2 $$\frac{\kappa }{{\kappa }_{P}}=\frac{{\tan }^{-1}(\beta )}{\beta }$$

where *κ*_*P*_ and *κ* are the lattice thermal conductivities of the parent and defected solids, respectively, and *β* is defined by:

3 $$\beta =(\frac{{\pi }^{2}{{\rm{\Theta }}}_{D}{\rm{\Omega }}}{h{\upsilon }_{m}^{2}}{\kappa }_{P}{\rm{\Gamma }})$$

where Ω is average volume per atom, *h* is Planck’s constant, Θ_*D*_ is Debye temperature, *υ*_*m*_ *is* average sound velocity and Γ is scattering coefficient. Scattering coefficient Γ is consist of two parts: one is mass fluctuation and the other is strain field fluctuation which is defined as:

4 $${\rm{\Gamma }}={x}_{i}[(\frac{{M}_{i}-\bar{M}}{\bar{M}})+\varepsilon {(\frac{{\delta }_{i}-\bar{\delta }}{\bar{\delta }})}^{2}]$$

where *x*_*i*_ is the concentration of defects, *M*_*i*_ is the atomic mass of the dopant, *δ*_*i*_ is the ionic radius of the dopant, $\bar{M}$ and $\bar{\delta }$ are the average atomic mass and mean ionic radius in the specific site in the solid solutions and *ε* is strain field factor. Therefore, we can find that the decrease of thermal conductivity of (Ho_x_Y_1-x_)_2_SiO_5_ mainly origin from the fluctuations of mass $(\frac{{M}_{i}-\bar{M}}{\bar{M}})$ and strain filed $(\frac{{\delta }_{i}-\bar{\delta }}{\bar{\delta }})$. The molar mass and ionic radius of Ho and Y are 164.9 and 88.9 g/mol, and 0.894 and 0.88 Å, respectively^8^. The large difference between molar mass and ionic radius would cause obvious lattice distortion and enhance phonon scattering, and finally reduce the thermal conductivity. Figure 4c shows the temperature dependent thermal conductivities of several promising TBC candidates^13^. The thermal conductivity of (Ho_1/3_Y_2/3_)_2_SiO_5_ and (Ho_2/3_Y_1/3_)_2_SiO_5_ are much lower than most of the candidates and they are closed to that of Gd_2_Zr_2_O_7_. The thermal conductivity of the widely used thermal barrier coating material 7YSZ is nearly 1 W/(m·K) higher than that of (Ho_1/3_Y_2/3_)_2_SiO_5_ and (Ho_2/3_Y_1/3_)_2_SiO_5_. Therefore, it is an efficient method to decrease the thermal conductivity Y_2_SiO_5_ by doping Ho.

**Figure 5:**
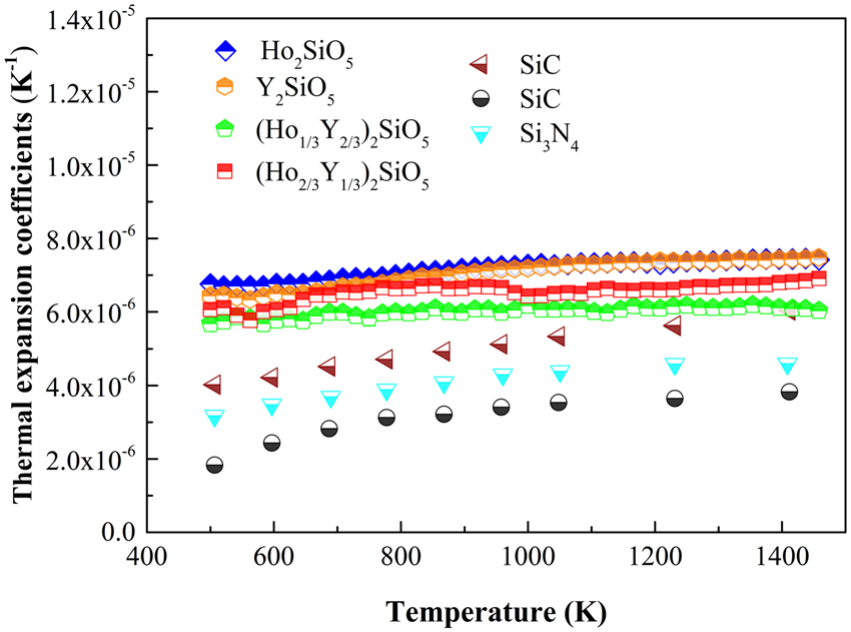
Thermal expansion coefficients of (Ho_x_Y_1-x_)_2_SiO_5_ and some Si-based ceramics.

Compatible thermal expansion coefficients with Si-based substrates is of great significance for ETBC. Figure 5 illustrates the temperature dependent thermal expansion coefficients of (Ho_x_Y_1-x_)_2_SiO_5_. The thermal expansion coefficients are shown from 500 K and the lower temperature values are omitted due to strain release between the samples and the test fixture^14^. Thermal expansion coefficients of Y_2_SiO_5_ and Ho_2_SiO_5_ are close and they are obviously larger than that of Si-based ceramics. Through doping with 2/3 Ho, thermal expansion coefficients can be slightly decreased, such as 6.86 × 10^−6^/K at 1473 K. When doped with 1/3 Ho, thermal expansions coefficient can be significantly reduced and are much closer to that of SiC ceramic, especially at high temperature, for instance, 5.89 × 10^−6^/K at 1473 K. Reduction of thermal expansion coefficients may originate from the doping induced distortion of crystal lattice. Thermal expansion of RE_2_SiO_5_ is a consequence of the anharmonicity of lattice vibrations and is related to the magnitude and sign of Grüneisen constants of phonons. Luo *et al*.^10^ found two groups of phonons in X2-Y_2_SiO_5_, one group with positive Grüneisen constants, and the other with negative Grüneisen constants. These two species of phonons contribute to positive and negative thermal expansion, respectively; and thermal expansion coefficient is a compromise of these two contributing contents. Li *et al*.^11^ also found that low-frequency phonons in X2-RE_2_SiO_5_ extensively have negative mode Grüneisen constants and these phonons contribute to negative thermal expansion. Doping in RE_2_SiO_5_ may cause more low-frequency phonons having negative mode Grüneisen constants and then contributing more to negative content of thermal expansion. As a result, macroscopic thermal expansion coefficients of solid solutions have smaller magnitude compared with two end members, Y_2_SiO_5_ and Ho_2_SiO_5_. The reduced thermal expansion coefficients of (Ho_1/3_Y_2/3_)_2_SiO_5_ makes it a promising candidate of ETBC.

ETBC are susceptible to the extremely high-temperature combustion environment. Degradation of the coating can occur by gradual thinning and erosion from the surface ultimately leading to complete failure. Visual examination through a borescope cannot identify such gradual degradation; only total delamination can be detected. Therefore, a quantitative nondestructive diagnostic approach should be developed to provide an adequate warning before the decreased environmental or thermal protection due to reductions in ETBC thickness or safety threatening thresholds^15^. Non-contact luminescence method was first put forward by Amano *et al*. to monitor the health of TBCs^16^. Trivalent lanthanide ions were introduced into the crystal structure, local information can be collected through conveyed by the luminescence emissions from optically excited luminescent layers. It is suggested that embedding luminescent sublayers into ETBCs to serve as erosion markers, so that when erosion exposes the luminescent “marker” layer, the luminescence characteristic of that layer will be produced when illuminated by the appropriate excitation wavelength^17^. To extend our knowledge of RE_2_SiO_5_ as self-monitoring ETBCs, the luminescence properties of (Ho_x_Y_1-x_)_2_SiO_5_ were comprehensively investigated.

**Figure 6:**
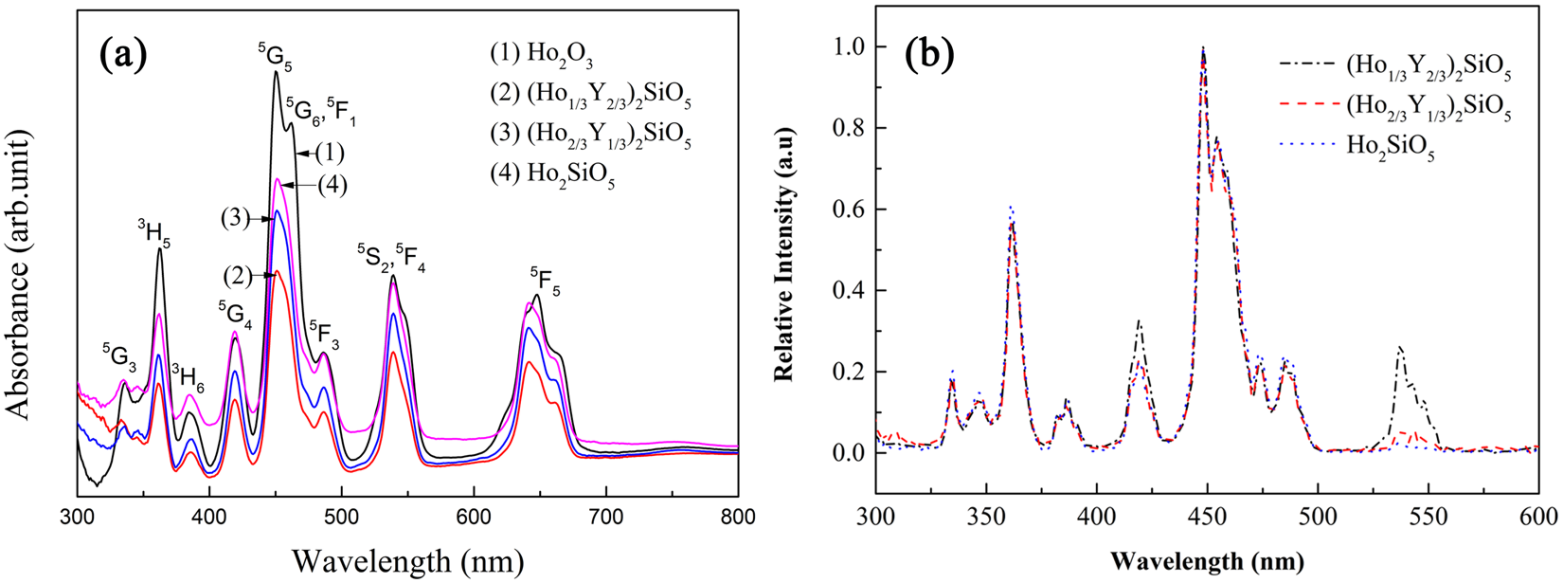
(**a**) Absorption and (**b**) excitation spectra of (Ho_x_Y_1-x_)_2_SiO_5_.

**Figure 7:**
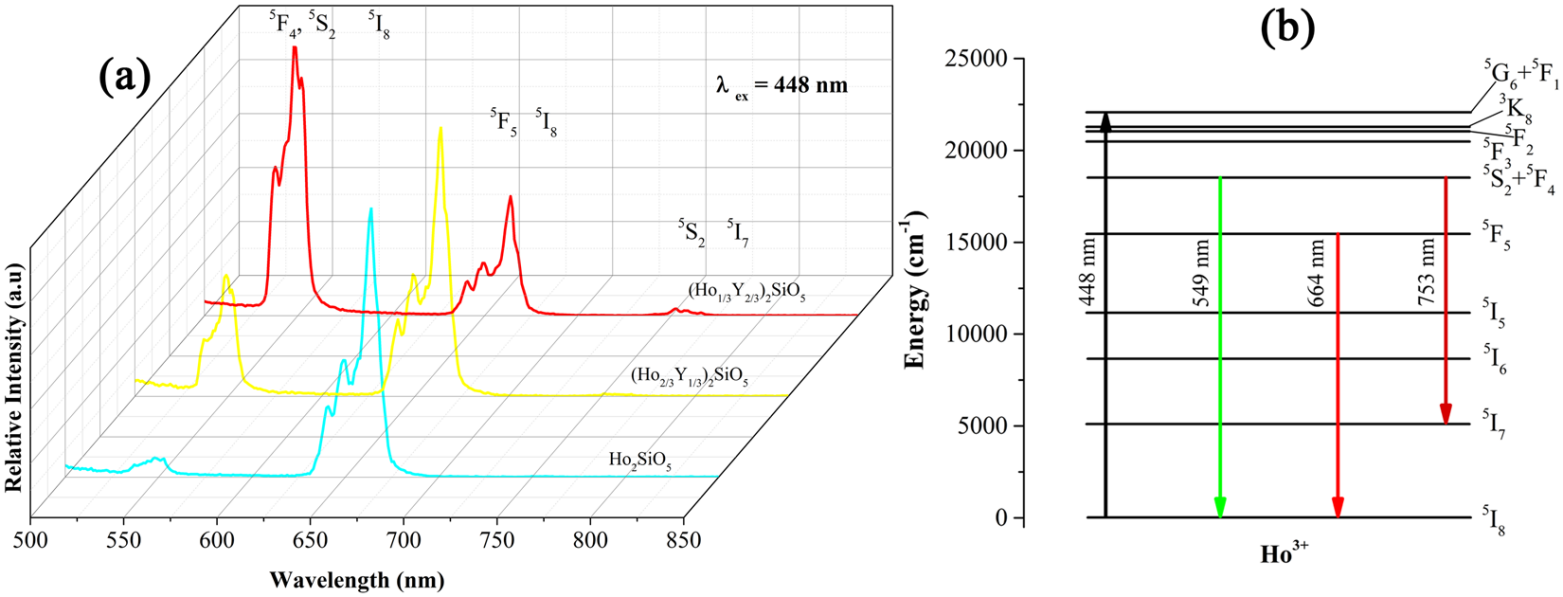
(**a**) Emission spectra and (**b**) energy level transition diagram of (Ho_x_Y_1-x_)_2_SiO_5_.

Figure 6a displays the UV-Vis absorption spectra of (Ho_1/3_Y_2/3_)_2_SiO_5_, (Ho_2/3_Y_1/3_)_2_SiO_5_, Ho_2_SiO_5_ and Ho_2_O_3_. They exhibit abundant peaks from 300 to 700 nm. The strongest peaks are located around 450 nm. The intensities of peaks are in direct proportion to the concentration of Ho. With the increase of Ho content, the intensities of peaks enhance gradually. Figure 6b shows the excitation spectra of (Ho_1/3_Y_2/3_)_2_SiO_5_, (Ho_2/3_Y_1/3_)_2_SiO_5_, and Ho_2_SiO_5_. They present the same excitation peaks and strong peaks appear at 448 nm. Photoluminescence spectra of (Ho_1/3_Y_2/3_)_2_SiO_5_, (Ho_2/3_Y_1/3_)_2_SiO_5_, and Ho_2_SiO_5_ were measured using 448 nm excitation light and displayed in Fig. 7. The main emission band of Ho_2_SiO_5_ is centered at 664 nm corresponding to the ^5^F_5_ to ^5^I_8_ transition. Another weak emission band is located at 549 nm induced by ^5^S_2_ + ^5^F_4_ to ^5^I_8_ transition. (Ho_2/3_Y_1/3_)_2_SiO_5_ contains two emission band, and emission band at 549 nm is stronger than that of Ho_2_SiO_5_. (Ho_1/3_Y_2/3_)_2_SiO_5_ also possesses two main emission bands. Emission band at 664 nm is weaker than those of (Ho_2/3_Y_1/3_)_2_SiO_5_ and Ho_2_SiO_5_ and it exhibits stronger emission band at 549 nm.

**Figure 8:**
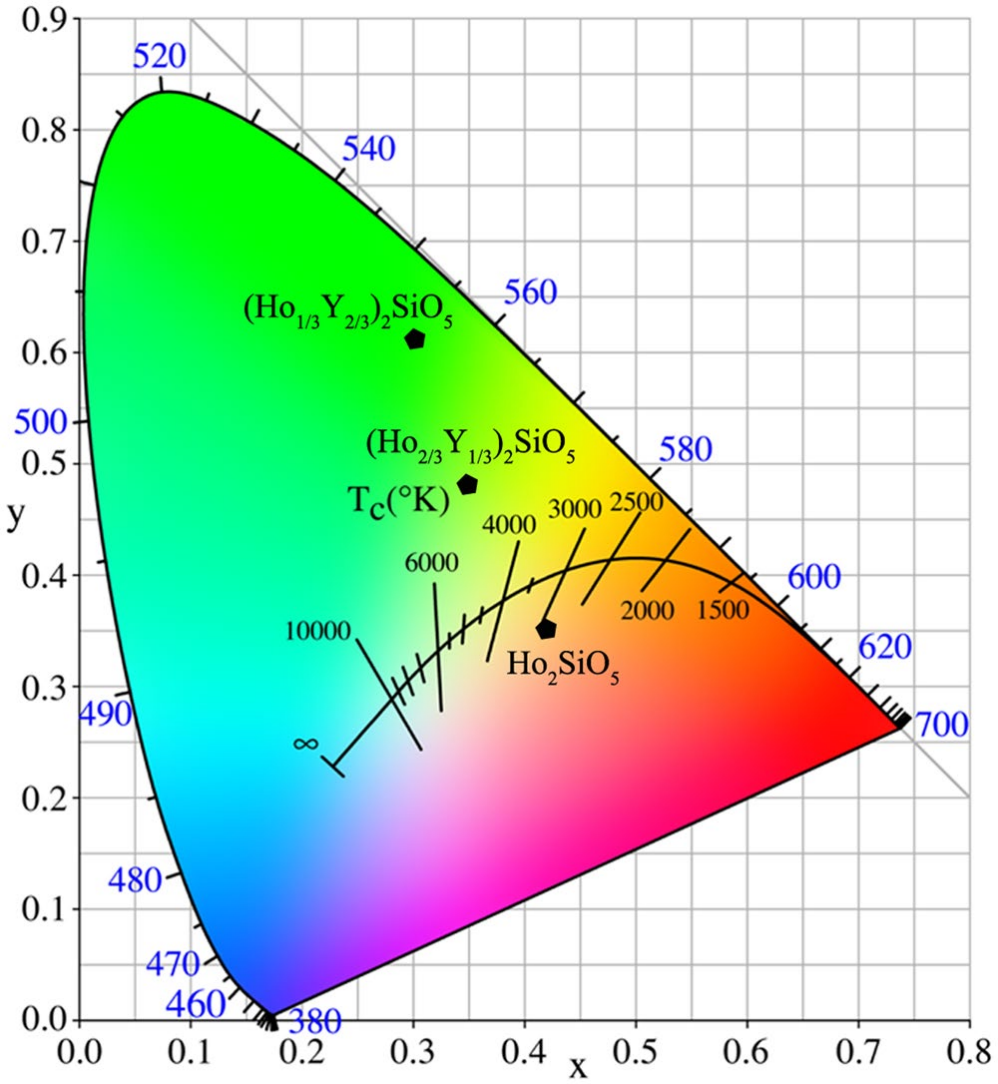
CIE chromaticity diagram of (H_1/3_Y_2/3_)_2_SiO_5_, (Ho_2/3_Y_1/3_)_2_SiO_5_ and Ho_2_SiO_5_.

The CIE (Commission Internationale de L’Eclairage) chromaticity diagram of (Ho_1/3_Y_2/3_)_2_SiO_5_, (Ho_2/3_Y_1/3_)_2_SiO_5_, Ho_2_SiO_5_ are presented in Fig. 8. With the increase of Ho concentration, luminescence color changes from green ((Ho_1/3_Y_2/3_)_2_SiO_5_) to yellow-green ((Ho_2/3_Y_1/3_)_2_SiO_5_), then to orange-red (Ho_2_SiO_5_). When (Ho_x_Y_1-x_)_2_SiO_5_ silicates are used as different players in ETBCs, quantitative assessment of coating thickness degradation and erosion can be made using these luminescent layers of known position and thickness. Therefore, through the design of Ho concentration in a multi-layered or gradient ETBC coating, the luminescence color can be modified in a wide range. Above all, (Ho_x_Y_1-x_)_2_SiO_5_ solid solutions with tunable luminescence color, low thermal conductivity, and matchable thermal expansion coefficients are novel damage self-monitoring ETBC candidates.

## Conclusions

(Ho_x_Y_1-x_)_2_SiO_5_ solid solutions are prepared by the hot pressing method. The impact of Ho substitution on the thermophysical properties of (Ho_x_Y_1-x_)_2_SiO_5_ solid solutions is investigated. Young’s modulus can be slightly decreased and high-temperature stiffness can be enhanced. The thermal conductivity of Y_2_SiO_5_ is significantly reduced by doping Ho due to the multiple enhanced phonon scattering mechanisms. Thermal expansion coefficients can also be modified closer to Si-based ceramics. In addition, (Ho_x_Y_1-x_)_2_SiO_5_ exhibits tunable luminescence colors. These silicates present green, to yellow-green, then to orange-red colors with increased Ho concentration. This work highlights (Ho_x_Y_1-x_)_2_SiO_5_ a novel damage self-monitoring ETBC candidates for Si-based ceramics.
